# The Cross-Talk between Spirochetal Lipoproteins and Immunity

**DOI:** 10.3389/fimmu.2014.00310

**Published:** 2014-06-30

**Authors:** Theodoros Kelesidis

**Affiliations:** ^1^Division of Infectious Diseases, Department of Medicine, David Geffen School of Medicine, University of California Los Angeles, Los Angeles, CA, USA

**Keywords:** spirochetes, lipoproteins, immunity, *Borrelia*, treponemes, lipopeptides, immunomodulation

## Abstract

Spirochetal diseases such as syphilis, Lyme disease, and leptospirosis are major threats to public health. However, the immunopathogenesis of these diseases has not been fully elucidated. Spirochetes interact with the host through various structural components such as lipopolysaccharides (LPS), surface lipoproteins, and glycolipids. Although spirochetal antigens such as LPS and glycolipids may contribute to the inflammatory response during spirochetal infections, spirochetes such as *Treponema pallidum* and *Borrelia burgdorferi* lack LPS. Lipoproteins are most abundant proteins that are expressed in all spirochetes and often determine how spirochetes interact with their environment. Lipoproteins are pro-inflammatory, may regulate responses from both innate and adaptive immunity and enable the spirochetes to adhere to the host or the tick midgut or to evade the immune system. However, most of the spirochetal lipoproteins have unknown function. Herein, the immunomodulatory effects of spirochetal lipoproteins are reviewed and are grouped into two main categories: effects related to immune evasion and effects related to immune activation. Understanding lipoprotein-induced immunomodulation will aid in elucidating innate immunopathogenesis processes and subsequent adaptive mechanisms potentially relevant to spirochetal disease vaccine development and to inflammatory events associated with spirochetal diseases.

## Introduction

Spirochetes are the cause of important human diseases such as syphilis, Lyme disease, and leptospirosis that are major threats to public health (1). However, the immunopathogenesis of these diseases has not been fully elucidated. Tissue inflammation is characteristic of spirochetal diseases such as dermatitis in syphilis and Lyme disease, interstitial nephritis in leptospirosis, and periodontitis caused by oral treponemes (2, 3). Spirochetes such as: *Treponema pallidum* (*T. pallidum*) and *Borrelia burgdorferi* (*B. burgdorferi*), the pathogens for syphilis and Lyme disease, respectively (1), may persist for prolonged periods despite the induced immune responses in the host (4–6). Several mechanisms may explain how spirochetes may evade host defenses such as intracellular sequestration of the spirochetes, the antigenic variation of the spirochetes, manipulation of host defenses to delay, and/or suppress the onset of effective immune responses and structural features of the outer membrane in spirochetes that contribute to immune evasion (7–11).

**Figure 1:**
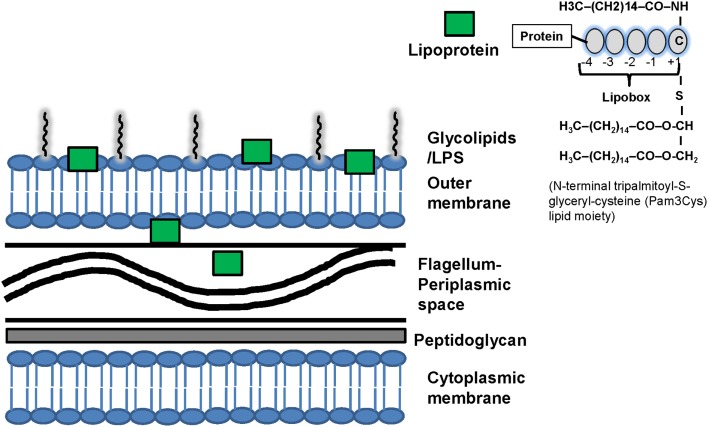
**Structure of spirochetal membrane and lipoproteins**. Similarly to gram-positive bacteria, the spirochetal cytoplasmic membrane is associated with the cell wall that consists of peptidoglycan. Similarly to Gram-negative bacteria, spirochetes also have an outer membrane, which is not attached to the peptidoglycan layer. Spirochetes differ phylogenetically from Gram-negative bacteria and interact with the host through various structural components such as lipopolysaccharides (LPS), surface lipoproteins and glycolipids that are present mostly in the outer membrane. LPS has not been identified in *Borrelia* and *Treponema*. The periplasmic space contains the flagellum. The distribution of lipoproteins varies among spirochetes and they may be present in different cellular compartments: the outer membrane, the extracellular and the periplasmic spaces. For example the pro-inflammatory lipoproteins of *T. pallidum* are located below its cell surface and thus do not interact directly with the immune system of the host. It has been suggested that uptake and degradation of *T. pallidum* releases lipoproteins and allows their interaction with receptors on immune cells leading to immune cell activation. Computational programs can predict spirochetal protein lipidation but do not determine the location of lipoproteins in the cells. Recently, developed fluorescence activated cell sorting (FACS) and surface proteolysis methods can be used to screen for lipoprotein localization. Right upper corner: structure of spirochetal lipoproteins. The finding of a cysteine residue after a signal peptide (+1) is suggestive evidence that a protein is lipidated. The spirochetal lipoproteins have a lipobox that is four amino acids in length and mediates NH2-terminal lipidation on a conserved cysteine residue. Lipoproteins interact with the phospholipids of membranes via three hydrophobic N-terminal acyl moieties (often palmitate; C16) attached to a N-terminal cysteine residue which may contribute to the localization of spirochetal lipoproteins. An analysis of the fatty acids of *T. pallidum*, *B. burgdorferi*, *L. interrogans* phospholipids and lipoproteins found that while fatty acids with different length side chains (C16 and C18) were found in phospholipids, palmitate (C16) predominated in the lipoproteins. The N-terminal tripalmitoyl-*S*-glyceryl-cysteine (Pam3Cys) lipid moiety is the part of the lipoprotein that confers its immunologic activity. C, cysteine; LPS, lipopolysaccharides.

Spirochetes have unique membrane structure that interacts with the immune system (Figure 1) (2, 3, 12–18). Although spirochetal antigens such as lipopolysaccharides (LPS), the main pro-inflammatory component of Gram-negative bacteria (19), and glycolipids may contribute to the inflammatory response during spirochetal infections, spirochetes such as *T. pallidum* and *B. burgdorferi* express abundantly membrane lipoproteins and induce strong immune responses (20–24) despite lack of LPS (2, 3, 17, 18). Thus, lipid–lipid interactions between spirochetes and the lipid rafts in eukaryotic host cells either through glycolipids (3, 25, 26) or lipoproteins (2, 18, 22, 27–31) may occur and these lipid interactions may be an important process that contributes to the immunopathogenesis of spirochetal diseases (3, 25, 26).

In bacteria, membrane lipoproteins are important virulence factors, pro-inflammatory agonists, enzymes, receptors, modular components of ATP binding cassette (ABC) transporters, and protective immune targets that regulate innate immunity (2, 3). In contrast to other bacteria that do not express lipoproteins so abundantly (2, 3, 27), lipoproteins have an important role in the virulence of spirochetes since they are most abundant proteins that are expressed in all spirochetes (2, 3, 18, 22, 27–31). The spirochetes express numerous lipoprotein genes [*T. pallidum* has >20 (24), *B. burgdorferi* has >100 lipoproteins (23), and approximately 8% of *B. burgdorferi* genes may encode lipoproteins (21, 23) and *Leptospira* spp. Have >140 lipoprotein genes (32)]. Examples of abundant lipoproteins in spirochetes include Tp47 of *T. pallidum*, OspA of *B. burgdorferi*, LipL32 of *Leptospira* species, and Vmp proteins of *Borrelia* species (Table 1). Finally, spirochetal lipoproteins have more prominent pro-inflammatory effects compared to other bacterial lipoproteins and synthetic lipopeptides (28).

**Table 1:** **Immunoregulatory effects of major known spirochetal lipoproteins**.

Spirochetal lipoproteins	Endothelial cells	Neutrophils	Complement	Antigen presenting cells: monocytes/macrophages/DCs	Lymphocytes
*Treponema pallidum*: mixture of bacterial lipoproteins of various MW [17 kDA (33), 38 kDA (34), 47 kDA (35)] and related synthetic lipopeptides (22, 30)	Activate directly host vascular endothelium which plays important roles in lymphocyte homing and hemostasis (36)	NR	NR	Stimulate macrophage and mDCs function: costimulatory signals (DC-SIGN, CD14) (11, 37, 38) and production of chemokines (CCR5) (39, 40), cytokines such as TNF-a, IL-1 beta, IL-6, and IL-12 (18, 33, 35, 41) through TLRs (42) and mostly TLR2 (43), activated IL-12 p40 promoter (42), NF-KB pathway (37, 38)	Up-regulate CCR5 expression on CD4+ T cells (39, 40)
*Borrelia burgdorferi:* outer-surface protein A (OspA) and B (OspB) and related synthetic lipopeptides (20, 44–46)	NR	OspB inhibits the phagocytosis and oxidative burst of human neutrophils whereas OspA induces the oxidative burst in neutrophils (47)	Deactivation of host complement by binding to CFH and FHL-1 (47–49)	Stimulate macrophage function and production of nitric oxide (42, 50), chemokines (CXCL13) (51), pro-inflammatory (such as TNF-a, IL-1 beta, IL-6, and IL-12) and anti-inflammatory (IL-10) cytokines (18, 35, 41, 44, 52–55) through TLRs (28, 42, 44, 45, 56, 57), CD14, and NF-kB activation pathway (37, 38, 44, 45). Also increase chemotaxis of circulating pDCs into skin (11) but do not activate pDCs *in vitro* and *in vivo* (58, 59)	Induce memory B cell immune responses (60), B cell proliferation and production of cytokines (61) and Th production of cytokines (IFN-γ and IL-6) (62) and chemokines (CXCL13) (51). OspA may bind TLR 2 and 6, activate NFκB and up-regulate costimulatory molecules as well as of MHC class II, leading to stronger T cell activation (63–65); Possible molecular mimicry for T helper cells between OspA-1 and LFA-1 (66–68). OspA-1 may activate autoreactive T cells against a self-epitope and adaptive immune responses to OspA are implicated in the pathogenesis of antibiotic- refractory Lyme arthritis (68, 69)
*Leptospira interrogans* LipL32 is the most abundant protein on the outer membrane of *Leptospira* and is expressed at high levels during infection (2, 70–76)	LipL32 interacts with endothelial cells contributing to systemic inflammation (77–80)	NR	NR	The calcium-binding cluster is crucial for the interaction between LipL32 and TLR2, which then triggers the signaling cascade of inflammatory responses (56, 72, 81)	Lipl32 has been used as immunogen for vaccine trials (82, 83)

*CFH, complement factor H; FHL-1, factor H-like protein 1; IL, interleukin; kDA, kilodalton; LipL32, 32-kDa lipoprotein of *Leptospira*; LFA-1, human lymphocyte function associated antigen 1; mDCs, myeloid dendritic cells; MW, molecular weight; NF-kB, NF-kappa B; NR, not reported; OspA, outer-surface protein A; OspB, outer-surface protein B; pDCs, plasmacytoid dendritic cells; TLR, toll-like receptor; Th, T helper; TNF-a, tumor necrosis factor*.

Surface-exposed lipoproteins often determine how spirochetes interact with their environment and immunity (Figure 1) (2, 3, 18, 22, 27–31). Lipoproteins may be present in different cellular compartments (2, 70, 84) and their distribution varies among spirochetes (7, 18, 85–88) (Figure 1). The NH2-terminal lipopeptide region is the part of the lipoprotein that confers its immunologic activity since removal of this lipid component removed the immunoregulatory properties of these lipoproteins while synthetic lipopeptides based on this lipid component could activate immune cells (Figure 1) (7–11, 18, 28, 89–93).

*In vitro* and *in vivo* studies suggest that spirochetal membrane lipoproteins and lipopeptides are pathogen-associated molecular patterns (PAMPs) that bind to pattern recognition receptors such as toll-like receptors (TLR1, 2). Thus, spirochetal lipoproteins are pro-inflammatory (18, 33, 35–37, 41, 94) by activating endothelial cells (36, 77–80, 95) and cells of innate immunity such as macrophages and dendritic cells (DCs) (7–11, 38, 42–45, 56, 63, 96, 97). Spirochetal lipoproteins also enable the spirochetes to evade the immune system (98, 99) and adhere to the host (100–104) or the tick midgut (105, 106) (Table 1). Finally, lipoproteins may be used as vaccine candidates for prevention of spirochetal infections (2, 3). The immunoregulatory effects of spirochetal lipoproteins have mostly been determined for major lipoproteins of *T. pallidum* (e.g., Tp47), *B. burgdorferi* (e.g., OspA, B), and *Leptospira* (e.g., LipL32) (Table 1). However, most spirochetal lipoproteins have not been well-studied and their interplay with immunity has recently been elucidated (Table S1 in Supplementary Material). Different methods have been used (Table 2) but observations in human models are often different from those obtained *in vitro*.

**Table 2:** **Methods used to determine *in vitro* and *in vivo* immunomodulatory properties of spirochetal lipopeptides/lipoproteins**.

Method	Comments
Mutagenesis systems	The lack of mutagenesis systems for *T. pallidum* significantly impairs studies of their immunopathogenesis Mutagenesis systems for *B. burgdorferi* have recently been developed (107
Structural studies	*T. pallidum* cannot be cultured in the laboratory and structural studies have been used to elucidate the function of treponemal lipoproteins (108)
Synthetic lipopeptides	The immunomodulatory properties of lipopeptides are conferred by the lipid moiety of their N termini and synthetic lipopeptides have been modeled after this structure to study native spirochetal lipoproteins (18, 33, 35–37, 41, 94) The synthetic lipopeptides have qualitatively similar immunostimulatory properties to those of native lipoproteins (18, 33, 35–37, 41, 94) Can be isolated in large amounts whereas large amounts of native lipoprotein cannot be isolated from spirochetes that cannot be cultured such as *T. pallidum* LPS contamination is a major problem when purifying bacterial lipoprotein while synthetic lipopeptides are synthesized under sterile conditions (22, 24, 35)
Skin techniques: injection of the skin with synthetic lipopeptides	Useful tools to study cellular responses induced by spirochetal lipopeptides within tissues Can be used instead of immunohistochemistry to characterize cellular infiltrates in target tissues of spirochetal disease (40, 109, 110)
Identification of stereotypical responses to lipopeptides *in vitro* (synthetic lipopeptides) and *in vivo* (presence of similar histopathological abnormalities)	Often contribute to the understanding of specific immunomodulatory effects of spirochetal lipoproteins (6, 18, 37, 50, 61, 94, 111)

*LPS, lipopolysaccharide*.

**Figure 2:**
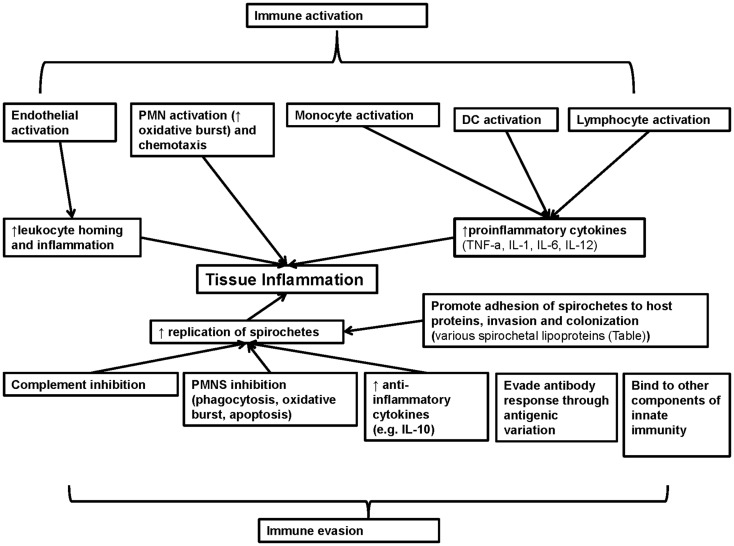
**Summary of the role of known spirochetal lipoproteins in regulation of immunity**. Spirochetal lipoproteins have two different major effects on immunity: immune evasion and immune activation. These lipoproteins may contribute to immune evasion through inhibition of complement, neutrophils, production of anti-inflammatory cytokines, evasion of antibody responses through antigenic variation and binding to other components of innate immunity (e.g., apolipoproteins). In addition, spirochetal lipoproteins may directly promote spirochetal tissue invasion and colonization and in combination with immune evasion may lead to increased spirochetal replication and tissue inflammation. Spirochetal lipoproteins may also directly and indirectly activate endothelial, epithelial cells, and immune cells that contribute to innate immune responses (neutrophils, monocytes, macrophages, and DCs) or adaptive immune responses (lymphocytes such as B cells and CD4 T helper cells). Collectively, these effects lead to increase inflammation in target tissues (e.g., skin) or adaptive autoimmune responses (e.g., arthritis) that contribute to the clinical manifestations of spirochetal diseases. IL, interleukin; TNF-a, tumor necrosis factor A.

Lipoproteins are environmentally regulated and may be expressed selectively during spirochetal infection (37, 50, 112–116) (Table 1 in Supplementary Material). For example, the outer-surface protein (Osp) A has a more important role in the pathogenesis of borrelial infection during the tick phase of *B. burgdorferi* and its expression is down regulated during the mammalian phase of *B. burgdorferi* infection (9). Thus, although OspA does not have a major role in regulation of host immunity *in vivo*, since it is not expressed during the later stages of borrelial infection, it has been used as a model to study *in vitro* the immunoregulatory effects of spirochetal lipoproteins (9). Herein, the immunomodulatory effects of spirochetal lipoproteins are reviewed and are grouped into two main categories: effects related to immune evasion and effects related to immune activation (Figure 2). Understanding these mechanisms will aid in elucidating the immunopathogenesis of chronic spirochetal diseases.

## The Role of Spirochetal Lipoproteins in Immune Evasion

Spirochetes may use outer membrane lipoproteins to invade the host but host immunity may also target these lipoproteins. However, all pathogenic spirochetes may cause persistent infections in humans by evading the host immune response through multiple mechanisms such as limiting the expression of membrane lipoproteins (85) and their access to antibodies (15, 117) and antigenic variation of surface lipoproteins (Table 1) (2, 98, 118–124). Spirochetal lipoproteins may also interact with and inhibit components of innate immunity such as the complement (10, 43, 56, 57, 97, 125–130), neutrophils, and serum lipoproteins (131).

### Spirochetes use antigenic variation of surface lipoproteins to evade immunity

Antigenic variation in borrelias may result from recombination of variable large and small protein genes (122) and the diversity of Vmp lipoproteins allows these pathogens to evade the host immune response (2, 3). Studies in immunocompromised hosts have suggested that the host immune responses have a major role in producing spirochetal antigenic variants (120).

### Spirochetal lipoproteins inhibit complement activation

Spirochetes may evade immune responses by inhibiting complement, a major innate immune system of the host (132, 133). Complement activation is caused by pathogen surface antigens such as LPS, antigen–antibody complexes, and binding of lectin to bacterial surfaces (134). Activation is regulated by host regulatory proteins, including factor H (FH) (134). *Borrelia* bind complement regulator FH and/or FH-like protein 1 (FHL-1) by directly interacting with Osp designated complement regulator-acquiring surface proteins (CRASPs) (135). Numerous surface spirochetal lipoproteins (outlined in Table 1; Table 1 in Supplementary Material; Figure 2) such as OspA, OspE, CspA may contribute to complement inhibition by binding to major complement regulatory proteins such as FH, FHL-1 C4b-binding protein (C4bp) and human C1 esterase inhibitor (C1-Inh) (48, 99, 136–147). Thus, spirochetal lipoproteins contribute to immune evasion through complement inhibition.

### Spirochetal lipoproteins may inhibit neutrophil function

Except for complement resistance, inhibition of neutrophil function is another mechanism that *B. burgdorferi* uses to evade the immune system. OspB inhibits the phagocytosis and oxidative burst of human neutrophils enabling *B. burgdorferi* to resist phagocytosis and oxidative burst in areas such as joint, skin, and the nervous system (47–49). Other spirochetal lipoproteins such as LIC11207 in *Leptospira* spp. may up-regulate the apoptosis of neutrophils (80). Thus, spirochetal lipoproteins may inhibit neutrophil function which may help the survival of the spirochetes.

## The Role of Spirochetal Lipoproteins in Immune Activation

### Spirochetal lipoproteins may activate neutrophils

Neutrophils are present in the joint fluid of Lyme arthritis patients (1), suggesting that they contribute to immune responses against *B. burgdorferi*. Although OspA and OspB are similarly regulated in the life cycle of *B. burgdorferi*, they have different functions. Contrary to OspB, OspA may activate human neutrophils (116) induce their oxidative burst (47) and induce neutrophil chemotaxis (148). OspA and OspC up-regulate complement receptor 3 (CR3), an adhesion molecule expressed on neutrophils that is involved in the interactions of *Borrelia* species with neutrophils (149), and OspA and OspB may bind to CR3 in a C3bi independent manner (150). Thus, different spirochetal lipoproteins may inhibit neutrophils to evade immune responses but others may activate neutrophils contributing to tissue inflammation.

### Spirochetal lipoproteins activate monocytes and macrophages to secrete cytokines through CD14 and TLR-dependent mechanisms

Bacterial lipoproteins and LPS both have an active lipid moiety and induce similar cell responses in similar cell types (19). CD14 is a protein in the membrane of macrophages that binds LPS and also induces lipoprotein signaling in several cells (38, 97, 151). Cellular membrane CD14 activates myeloid cells such as monocyte, macrophages, and polymorphonuclear white blood cells while soluble CD14 activates non-myeloid (endothelial, epithelial) cells (152). Spirochetal lipoproteins (such *T. pallidum* lipoproteins) bind to CD14 at the site that binds LPS and may activate monocytes and the NF-κB pathway through CD14 (38, 44, 97). In contrast to Gram-negative bacteria, LPS-binding protein (LBP) does not mediate interaction of spirochetal lipoproteins with CD14 (3, 38). However, TLR knockout and overexpression studies have confirmed that lipoproteins drive inflammation in syphilis and Lyme disease through TLR-dependent (TLR1, TLR2) responses (10, 43, 56, 57, 97, 125–130). Thus, during spirochetemia in Lyme disease and syphilis, spirochetal lipoproteins activate cells via TLR1 and TLR2 in contrast to Gram-negative sepsis where cellular activation occurs through TLR4 (45, 56, 63). Integrin α3β1 may co-operate with TLR2/TLR1 in mediating pro-inflammatory responses in human macrophages stimulated with spirochetal lipopeptides such as BBB07 (153, 154). However, TLR-independent receptor responses are also important for spirochetal induced inflammation (125, 154–156). *T. pallidum* lipoproteins, the *B. burgdorferi* OspA lipoprotein and synthetic lipopetides may up-regulate macrophage production of pro-inflammatory cytokines such as TNF-α, IL-1, IL-6, and IL-12 (18, 50). Many different cell types may produce IL-10 in response to stimulation by *B. burgdorferi* lipoproteins (52, 53, 62, 157, 158). On the other hand, endogenously produced and exogenous IL-10 significantly reduced OspA lipoprotein-induced macrophage production of cytokines and chemokines (54, 62, 158–161), consistently with previous studies that have shown that IL-10 may down-regulate the TLR signaling pathway (159). Thus, spirochetal lipoproteins induce immune responses in antigen presenting cells such as monocytes and macrophages through TLR-dependent and -independent mechanisms.

#### Spirochetal lipoproteins activate dendritic cells

Except for monocytes and macrophages, spirochetal lipoproteins may also activate other antigen presenting cells such as DCs. DCs are a major link between innate and adaptive immunity, since after activation, they up-regulate costimulatory molecules such as CD54 that interact with T cell receptors such as CD11a/CD18 within lymph nodes (162, 163). Consistent with the hypothesis that lipoproteins are key pro-inflammatory mediators in spirochetal diseases, many studies have shown that treponemal lipoproteins and synthetic lipopeptides can up-regulate CD54 and contribute to DC activation (18, 33, 35–37, 41, 94). Phagocytosis of intact spirochetes, activation of TLRs at phagosomes and bacterial cell death may result in the release of treponemal lipoproteins and may also contribute to immune cell activation (163). Also *B. burgdorferi* lipoproteins increase chemotaxis of circulating plasmacytoid dendritic cells (pDCs) into skin (11) but do not activate pDCs *in vitro* and *in vivo* (58, 59).

### Spirochetal lipoproteins induce inflammatory infiltrate into target tissues and adaptive immune responses *in vivo* that contribute to clinical manifestations of spirochetal diseases

Although lipoproteins may activate neutrophils, macrophages, endothelial cells *in vitro*, they may also induce inflammatory infiltrate into target tissues during spirochetal infection *in vivo* (37, 50, 61, 94, 115, 116, 164). Lipoproteins may also contribute to the pathogenesis of the Jarisch-Herxheimer reaction, a transient immunological phenomenon that occurs during treatment of spirochetal infections (165, 166). Injection of synthetic lipopeptides into the skin can also be used to study the immunomodulatory effects of spirochetal lipoproteins *in vivo* (94). Cutaneous injection of spirochetal lipopeptides and spirochetal skin infections elicit similar cellular infiltrate supporting the hypothesis that spirochetal lipoproteins recruit diverse leukocytes from peripheral blood into target tissues (10). Lipoprotein-responsive cells (167), such as endothelium (95, 168), keratinocytes (169), and macrophages (170) induce chemotaxis of mixed cellular infiltrate that in combination with extravasating leukocytes further increase the tissue inflammatory response. Spirochetal lipoproteins activate *in vivo* antigen presenting cells (macrophages, DCs, CD4+ T cells) within the inflamed skin in spirochetal diseases (39, 40, 58, 59). Lipopeptides in combination with other antigens from spirochetes facilitate the transition from innate to prolonged adaptive immune responses that contribute to chronic manifestations of spirochetal diseases such as syphilis and Lyme disease (11). Consistent with these data from *in vivo* studies, *in vitro* studies have demonstrated that spirochetal lipoproteins may directly activate both B and T cells (Table 1). These lipoprotein-induced adaptive immune responses may trigger autoimmune and vaccine immune responses (Table 1). Thus, spirochetal lipoproteins induce initially innate immunity and then adaptive immunity through recruitment of spirochete-specific T cells and tissue inflammation that is associated with clinical manifestations of spirochetal disease such as Lyme arthritis (37, 50, 94, 115, 116, 164).

## Conclusion

Lipoproteins are widely expressed by many pathogens and have pro-inflammatory effects (171–223). Thus, an understanding of how lipoproteins interact with the immune system will aid in the understanding of the pathogenesis of many infections including spirochetal infections. In addition, elucidating the molecular mechanisms of lipoprotein-induced immunomodulation (summarized in Figure 2) will lead to a greater understanding of the inflammatory processes, innate and adaptive immune responses associated with spirochetal diseases that may contribute to spirochetal disease vaccine development (163).

## Conflict of Interest Statement

The author declares that the research was conducted in the absence of any commercial or financial relationships that could be construed as a potential conflict of interest.

## Supplementay Material

The Supplementary Material for this article can be found online at http://www.frontiersin.org/Journal/10.3389/fimmu.2014.00310/abstract

## References

[1] Steere AC Lyme disease. N Engl J Med (2001) 345(2):115–25 10.1056/NEJM200107123450207 11450660

[2] Haake DA Spirochaetal lipoproteins and pathogenesis. Microbiology (2000) 146(Pt 7):1491–504 10878114 10.1099/00221287-146-7-1491 PMC2664406

[3] Schroder NW Eckert J Stubs G Schumann RR Immune responses induced by spirochetal outer membrane lipoproteins and glycolipids. Immunobiology (2008) 213(3–4):329–40 10.1016/j.imbio.2007.11.003 18406378

[4] Steere AC Coburn J Glickstein L The emergence of Lyme disease. J Clin Invest (2004) 113(8):1093–101 10.1172/JCI21681 15085185 PMC385417

[5] Hook EW III Peeling RW Syphilis control – a continuing challenge. N Engl J Med (2004) 351(2):122–4 10.1056/NEJMp048126 15247352

[6] Duray PH Histopathology of clinical phases of human Lyme disease. Rheum Dis Clin North Am (1989) 15(4):691–710 2685926

[7] Radolf JD Role of outer membrane architecture in immune evasion by *Treponema pallidum* and *Borrelia burgdorferi*. Trends Microbiol (1994) 2(9):307–11 10.1016/0966-842X(94)90446-4 7812663

[8] Radolf JD Desrosiers DC *Treponema pallidum*, the stealth pathogen, changes, but how? Mol Microbiol (2009) 72(5):1081–6 10.1111/j.1365-2958.2009.06711.x 19432802 PMC2975512

[9] Radolf JD Caimano MJ Stevenson B Hu LT Of ticks, mice and men: understanding the dual-host lifestyle of Lyme disease spirochaetes. Nat Rev Microbiol (2012) 10(2):87–99 10.1038/nrmicro2714 22230951 PMC3313462

[10] Salazar JC Hazlett KR Radolf JD The immune response to infection with *Treponema pallidum*, the stealth pathogen. Microbes Infect (2002) 4(11):1133–40 10.1016/S1286-4579(02)01638-6 12361913

[11] Salazar JC Pope CD Moore MW Pope J Kiely TG Radolf JD Lipoprotein-dependent and -independent immune responses to spirochetal infection. Clin Diagn Lab Immunol (2005) 12(8):949–58 10.1128/CDLI.12.8.949-958.2005 16085913 PMC1182186

[12] Paster BJ Dewhirst FE Weisburg WG Tordoff LA Fraser GJ Hespell RB Phylogenetic analysis of the spirochetes. J Bacteriol (1991) 173(19):6101–9 1917844 10.1128/jb.173.19.6101-6109.1991 PMC208357

[13] Barbour AG Hayes SF Biology of *Borrelia* species. Microbiol Rev (1986) 50(4):381–400 3540570 10.1128/mr.50.4.381-400.1986 PMC373079

[14] Takayama K Rothenberg RJ Barbour AG Absence of lipopolysaccharide in the Lyme disease spirochete, *Borrelia burgdorferi*. Infect Immun (1987) 55(9):2311–3 3623705 10.1128/iai.55.9.2311-2313.1987 PMC260699

[15] Cox DL Chang P McDowall AW Radolf JD The outer membrane, not a coat of host proteins, limits antigenicity of virulent *Treponema pallidum*. Infect Immun (1992) 60(3):1076–83 1541522 10.1128/iai.60.3.1076-1083.1992 PMC257596

[16] Hossain H Wellensiek HJ Geyer R Lochnit G Structural analysis of glycolipids from *Borrelia burgdorferi*. Biochimie (2001) 83(7):683–92 10.1016/S0300-9084(01)01296-2 11522398

[17] Rietschel ET Schletter J Weidemann B El-Samalouti V Mattern T Zahringer U Lipopolysaccharide and peptidoglycan: CD14-dependent bacterial inducers of inflammation. Microb Drug Resist (1998) 4(1):37–44 10.1089/mdr.1998.4.37 9533723

[18] Radolf JD Arndt LL Akins DR Curetty LL Levi ME Shen Y *Treponema pallidum* and *Borrelia burgdorferi* lipoproteins and synthetic lipopeptides activate monocytes/macrophages. J Immunol (1995) 154(6):2866–77 7876555

[19] Ulevitch RJ Tobias PS Recognition of Gram-negative bacteria and endotoxin by the innate immune system. Curr Opin Immunol (1999) 11(1):19–22 10.1016/S0952-7915(99)80004-1 10047547

[20] Brandt ME Riley BS Radolf JD Norgard MV Immunogenic integral membrane proteins of *Borrelia burgdorferi* are lipoproteins. Infect Immun (1990) 58(4):983–91 2318538 10.1128/iai.58.4.983-991.1990 PMC258571

[21] Casjens S Palmer N van VR Huang WM Stevenson B Rosa P A bacterial genome in flux: the twelve linear and nine circular extrachromosomal DNAs in an infectious isolate of the Lyme disease spirochete *Borrelia burgdorferi*. Mol Microbiol (2000) 35(3):490–516 10.1046/j.1365-2958.2000.01698.x 10672174

[22] Chamberlain NR Brandt ME Erwin AL Radolf JD Norgard MV Major integral membrane protein immunogens of *Treponema pallidum* are proteolipids. Infect Immun (1989) 57(9):2872–7 2668191 10.1128/iai.57.9.2872-2877.1989 PMC313540

[23] Fraser CM Casjens S Huang WM Sutton GG Clayton R Lathigra R Genomic sequence of a Lyme disease spirochaete, *Borrelia burgdorferi*. Nature (1997) 390(6660):580–6 10.1038/37551 9403685

[24] Fraser CM Norris SJ Weinstock GM White O Sutton GG Dodson R Complete genome sequence of *Treponema pallidum*, the syphilis spirochete. Science (1998) 281(5375):375–88 10.1126/science.281.5375.375 9665876

[25] LaRocca TJ Crowley JT Cusack BJ Pathak P Benach J London E Cholesterol lipids of *Borrelia burgdorferi* form lipid rafts and are required for the bactericidal activity of a complement-independent antibody. Cell Host Microbe (2010) 8(4):331–42 10.1016/j.chom.2010.09.001 20951967 PMC3010898

[26] Crowley JT Toledo AM LaRocca TJ Coleman JL London E Benach JL Lipid exchange between *Borrelia burgdorferi* and host cells. PLoS Pathog (2013) 9(1):e1003109 10.1371/journal.ppat.1003109 23326230 PMC3542181

[27] Liang FT Nelson FK Fikrig E DNA microarray assessment of putative *Borrelia burgdorferi* lipoprotein genes. Infect Immun (2002) 70(6):3300–3 10.1128/IAI.70.6.3300-3303.2002 12011030 PMC128019

[28] Weis JJ Ma Y Erdile LF Biological activities of native and recombinant *Borrelia burgdorferi* outer surface protein A: dependence on lipid modification. Infect Immun (1994) 62(10):4632–6 7927731 10.1128/iai.62.10.4632-4636.1994 PMC303154

[29] Braun V Hantke K Biochemistry of bacterial cell envelopes. Annu Rev Biochem (1974) 43(0):89–121 10.1146/annurev.bi.43.070174.000513 4277378

[30] Radolf JD Chamberlain NR Clausell A Norgard MV Identification and localization of integral membrane proteins of virulent *Treponema pallidum* subsp. pallidum by phase partitioning with the nonionic detergent triton X-114. Infect Immun (1988) 56(2):490–8 3276627 10.1128/iai.56.2.490-498.1988 PMC259309

[31] Bricker TM Boyer MJ Keith J Watson-McKown R Wise KS Association of lipids with integral membrane surface proteins of *Mycoplasma hyorhinis*. Infect Immun (1988) 56(2):295–301 3338843 10.1128/iai.56.2.295-301.1988 PMC259279

[32] Setubal JC Reis M Matsunaga J Haake DA Lipoprotein computational prediction in spirochaetal genomes. Microbiology (2006) 152(Pt 1):113–21 10.1099/mic.0.28317-0 16385121 PMC2667199

[33] Akins DR Purcell BK Mitra MM Norgard MV Radolf JD Lipid modification of the 17-kilodalton membrane immunogen of *Treponema pallidum* determines macrophage activation as well as amphiphilicity. Infect Immun (1993) 61(4):1202–10 8454324 10.1128/iai.61.4.1202-1210.1993 PMC281349

[34] Richarme G Kohiyama M Purification of the MglC/E membrane proteins of the binding protein-dependent galactose transport system of *Salmonella typhimurium*. FEBS Lett (1992) 304(2–3):167–9 10.1016/0014-5793(92)80611-J 1618318

[35] DeOgny L Pramanik BC Arndt LL Jones JD Rush J Slaughter CA Solid-phase synthesis of biologically active lipopeptides as analogs for spirochetal lipoproteins. Pept Res (1994) 7(2):91–7 8012126

[36] Riley BS Oppenheimer-Marks N Hansen EJ Radolf JD Norgard MV Virulent *Treponema pallidum* activates human vascular endothelial cells. J Infect Dis (1992) 165(3):484–93 10.1093/infdis/165.3.484 1347056

[37] Norgard MV Arndt LL Akins DR Curetty LL Harrich DA Radolf JD Activation of human monocytic cells by *Treponema pallidum* and *Borrelia burgdorferi* lipoproteins and synthetic lipopeptides proceeds via a pathway distinct from that of lipopolysaccharide but involves the transcriptional activator NF-kappa B. Infect Immun (1996) 64(9):3845–52 8751937 10.1128/iai.64.9.3845-3852.1996 PMC174301

[38] Sellati TJ Bouis DA Kitchens RL Darveau RP Pugin J Ulevitch RJ *Treponema pallidum* and *Borrelia burgdorferi* lipoproteins and synthetic lipopeptides activate monocytic cells via a CD14-dependent pathway distinct from that used by lipopolysaccharide. J Immunol (1998) 160(11):5455–64 9605148

[39] Sellati TJ Wilkinson DA Sheffield JS Koup RA Radolf JD Norgard MV Virulent *Treponema pallidum*, lipoprotein, and synthetic lipopeptides induce CCR5 on human monocytes and enhance their susceptibility to infection by human immunodeficiency virus type 1. J Infect Dis (2000) 181(1):283–93 10.1086/315209 10608777

[40] Sellati TJ Waldrop SL Salazar JC Bergstresser PR Picker LJ Radolf JD The cutaneous response in humans to *Treponema pallidum* lipoprotein analogues involves cellular elements of both innate and adaptive immunity. J Immunol (2001) 166(6):4131–40 10.4049/jimmunol.166.6.4131 11238663

[41] Radolf JD Norgard MV Brandt ME Isaacs RD Thompson PA Beutler B Lipoproteins of *Borrelia burgdorferi* and *Treponema pallidum* activate cachectin/tumor necrosis factor synthesis. Analysis using a CAT reporter construct. J Immunol (1991) 147(6):1968–74 1890308

[42] Brightbill HD Libraty DH Krutzik SR Yang RB Belisle JT Bleharski JR Host defense mechanisms triggered by microbial lipoproteins through toll-like receptors. Science (1999) 285(5428):732–6 10.1126/science.285.5428.732 10426995

[43] Lien E Sellati TJ Yoshimura A Flo TH Rawadi G Finberg RW Toll-like receptor 2 functions as a pattern recognition receptor for diverse bacterial products. J Biol Chem (1999) 274(47):33419–25 10.1074/jbc.274.47.33419 10559223

[44] Giambartolomei GH Dennis VA Lasater BL Philipp MT Induction of pro- and anti-inflammatory cytokines by *Borrelia burgdorferi* lipoproteins in monocytes is mediated by CD14. Infect Immun (1999) 67(1):140–7 9864208 10.1128/iai.67.1.140-147.1999 PMC96289

[45] Hirschfeld M Kirschning CJ Schwandner R Wesche H Weis JH Wooten RM Cutting edge: inflammatory signaling by *Borrelia burgdorferi* lipoproteins is mediated by toll-like receptor 2. J Immunol (1999) 163(5):2382–6 10452971

[46] Bergstrom S Bundoc VG Barbour AG Molecular analysis of linear plasmid-encoded major surface proteins, OspA and OspB, of the Lyme disease spirochaete *Borrelia burgdorferi*. Mol Microbiol (1989) 3(4):479–86 10.1111/j.1365-2958.1989.tb00194.x 2761388

[47] Hartiala P Hytonen J Suhonen J Lepparanta O Tuominen-Gustafsson H Viljanen MK *Borrelia burgdorferi* inhibits human neutrophil functions. Microbes Infect (2008) 10(1):60–8 10.1016/j.micinf.2007.10.004 18068388

[48] van Burgel ND Kraiczy P Schuijt TJ Zipfel PF van Dam AP Identification and functional characterisation of complement regulator acquiring surface protein-1 of serum resistant *Borrelia garinii* OspA serotype 4. BMC Microbiol (2010) 10:43 10.1186/1471-2180-10-43 20146822 PMC2833144

[49] Sadziene A Thomas DD Barbour AG *Borrelia burgdorferi* mutant lacking Osp: biological and immunological characterization. Infect Immun (1995) 63(4):1573–80 7890424 10.1128/iai.63.4.1573-1580.1995 PMC173191

[50] Ma Y Seiler KP Tai KF Yang L Woods M Weis JJ Outer surface lipoproteins of *Borrelia burgdorferi* stimulate nitric oxide production by the cytokine-inducible pathway. Infect Immun (1994) 62(9):3663–71 7520417 10.1128/iai.62.9.3663-3671.1994 PMC303016

[51] Rupprecht TA Kirschning CJ Popp B Kastenbauer S Fingerle V Pfister HW *Borrelia garinii* induces CXCL13 production in human monocytes through toll-like receptor 2. Infect Immun (2007) 75(9):4351–6 10.1128/IAI.01642-06 17562761 PMC1951179

[52] Giambartolomei GH Dennis VA Philipp MT *Borrelia burgdorferi* stimulates the production of interleukin-10 in peripheral blood mononuclear cells from uninfected humans and rhesus monkeys. Infect Immun (1998) 66(6):2691–7 9596735 10.1128/iai.66.6.2691-2697.1998 PMC108257

[53] Giambartolomei GH Dennis VA Lasater BL Murthy PK Philipp MT Autocrine and exocrine regulation of interleukin-10 production in THP-1 cells stimulated with *Borrelia burgdorferi* lipoproteins. Infect Immun (2002) 70(4):1881–8 10.1128/IAI.70.4.1881-1888.2002 11895951 PMC127882

[54] Brown JP Zachary JF Teuscher C Weis JJ Wooten RM Dual role of interleukin-10 in murine Lyme disease: regulation of arthritis severity and host defense. Infect Immun (1999) 67(10):5142–50 10496888 10.1128/iai.67.10.5142-5150.1999 PMC96863

[55] Diterich I Harter L Hassler D Wendel A Hartung T Modulation of cytokine release in ex vivo-stimulated blood from borreliosis patients. Infect Immun (2001) 69(2):687–94 10.1128/IAI.69.2.687-694.2001 11159956 PMC97940

[56] Alexopoulou L Thomas V Schnare M Lobet Y Anguita J Schoen RT Hyporesponsiveness to vaccination with *Borrelia burgdorferi* OspA in humans and in TLR1- and TLR2-deficient mice. Nat Med (2002) 8(8):878–84 10.1038/nm732 12091878

[57] Dennis VA Dixit S O’Brien SM Alvarez X Pahar B Philipp MT Live *Borrelia burgdorferi* spirochetes elicit inflammatory mediators from human monocytes via the toll-like receptor signaling pathway. Infect Immun (2009) 77(3):1238–45 10.1128/IAI.01078-08 19139200 PMC2643645

[58] Jarrossay D Napolitani G Colonna M Sallusto F Lanzavecchia A Specialization and complementarity in microbial molecule recognition by human myeloid and plasmacytoid dendritic cells. Eur J Immunol (2001) 31(11):3388–93 10.1002/1521-4141(200111)31:11<3388::AID-IMMU3388>3.0.CO;2-Q 11745357

[59] Kadowaki N Ho S Antonenko S Malefyt RW Kastelein RA Bazan F Subsets of human dendritic cell precursors express different toll-like receptors and respond to different microbial antigens. J Exp Med (2001) 194(6):863–9 10.1084/jem.194.6.863 11561001 PMC2195968

[60] Hogenesch H Mechanisms of stimulation of the immune response by aluminum adjuvants. Vaccine (2002) 20(Suppl 3):S34–9 10.1016/S0264-410X(02)00169-X 12184362

[61] Ma Y Weis JJ *Borrelia burgdorferi* outer surface lipoproteins OspA and OspB possess B-cell mitogenic and cytokine-stimulatory properties. Infect Immun (1993) 61(9):3843–53 8359905 10.1128/iai.61.9.3843-3853.1993 PMC281085

[62] Ganapamo F Dennis VA Philipp MT Early induction of gamma interferon and interleukin-10 production in draining lymph nodes from mice infected with *Borrelia burgdorferi*. Infect Immun (2000) 68(12):7162–5 10.1128/IAI.68.12.7162-7165.2000 11083848 PMC97833

[63] Aliprantis AO Yang RB Mark MR Suggett S Devaux B Radolf JD Cell activation and apoptosis by bacterial lipoproteins through toll-like receptor-2. Science (1999) 285(5428):736–9 10.1126/science.285.5428.736 10426996

[64] Bulut Y Faure E Thomas L Equils O Arditi M Cooperation of toll-like receptor 2 and 6 for cellular activation by soluble tuberculosis factor and *Borrelia burgdorferi* outer surface protein A lipoprotein: role of toll-interacting protein and IL-1 receptor signaling molecules in Toll-like receptor 2 signaling. J Immunol (2001) 167(2):987–94 10.4049/jimmunol.167.2.987 11441107

[65] Hertz CJ Kiertscher SM Godowski PJ Bouis DA Norgard MV Roth MD Microbial lipopeptides stimulate dendritic cell maturation via toll-like receptor 2. J Immunol (2001) 166(4):2444–50 10.4049/jimmunol.166.4.2444 11160304

[66] Gross DM Forsthuber T Tary-Lehmann M Etling C Ito K Nagy ZA Identification of LFA-1 as a candidate autoantigen in treatment-resistant Lyme arthritis. Science (1998) 281(5377):703–6 10.1126/science.281.5377.703 9685265

[67] Poland GA Vaccines against Lyme disease: what happened and what lessons can we learn? Clin Infect Dis (2011) 52(Suppl 3):s253–8 10.1093/cid/ciq116 21217172

[68] Steere AC Drouin EE Glickstein LJ Relationship between immunity to *Borrelia burgdorferi* outer-surface protein A (OspA) and Lyme arthritis. Clin Infect Dis (2011) 52(Suppl 3):s259–65 10.1093/cid/ciq117 21217173 PMC3106239

[69] Drouin EE Glickstein L Kwok WW Nepom GT Steere AC Human homologues of a *Borrelia* T cell epitope associated with antibiotic-refractory Lyme arthritis. Mol Immunol (2008) 45(1):180–9 10.1016/j.molimm.2007.04.017 17555819 PMC2075570

[70] Haake DA Matsunaga J *Leptospira*: a spirochaete with a hybrid outer membrane. Mol Microbiol (2010). 10.1111/j.1365-2958.2010.07262.x 20598085 PMC2976823

[71] Flannery B Costa D Carvalho FP Guerreiro H Matsunaga J Da Silva ED Evaluation of recombinant *Leptospira* antigen-based enzyme-linked immunosorbent assays for the serodiagnosis of leptospirosis. J Clin Microbiol (2001) 39(9):3303–10 10.1128/JCM.39.9.3303-3310.2001 11526167 PMC88335

[72] Lo YY Hsu SH Ko YC Hung CC Chang MY Hsu HH Essential calcium-binding cluster of *Leptospira* LipL32 protein for inflammatory responses through the toll-like receptor 2 pathway. J Biol Chem (2013) 288(17):12335–44 10.1074/jbc.M112.418699 23486465 PMC3636917

[73] Pinne M Haake DA LipL32 is a subsurface lipoprotein of *Leptospira interrogans*: presentation of new data and reevaluation of previous studies. PLoS One (2013) 8(1):e51025 10.1371/journal.pone.0051025 23323152 PMC3544172

[74] Malmstrom J Beck M Schmidt A Lange V Deutsch EW Aebersold R Proteome-wide cellular protein concentrations of the human pathogen *Leptospira interrogans*. Nature (2009) 460(7256):762–5 10.1038/nature08184 19606093 PMC2723184

[75] Cullen PA Xu X Matsunaga J Sanchez Y Ko AI Haake DA Surfaceome of *Leptospira* spp. Infect Immun (2005) 73(8):4853–63 10.1128/IAI.73.8.4853-4863.2005 16040999 PMC1201201

[76] Haake DA Matsunaga J Characterization of the leptospiral outer membrane and description of three novel leptospiral membrane proteins. Infect Immun (2002) 70(9):4936–45 10.1128/IAI.70.9.4936-4945.2002 12183539 PMC128291

[77] Sun Z Bao L Li D Huang B Wu B Effect of *Leptospira interrogans* outer membrane proteins LipL32 on HUVEC. Microb Pathog (2010) 49(3):116–21 10.1016/j.micpath.2010.05.006 20510346

[78] Atzingen MV Gomez RM Schattner M Pretre G Goncales AP de Morais ZM Lp95, a novel leptospiral protein that binds extracellular matrix components and activates e-selectin on endothelial cells. J Infect (2009) 59(4):264–76 10.1016/j.jinf.2009.07.010 19665803

[79] Gomez RM Vieira ML Schattner M Malaver E Watanabe MM Barbosa AS Putative outer membrane proteins of *Leptospira interrogans* stimulate human umbilical vein endothelial cells (HUVECS) and express during infection. Microb Pathog (2008) 45(5–6):315–22 10.1016/j.micpath.2008.08.004 18778767

[80] Pretre G Lapponi MJ Atzingen MV Schattner M Nascimento AL Gomez RM Characterization of LIC11207, a novel leptospiral protein that is recognized by human convalescent sera and prevents apoptosis of polymorphonuclear leukocytes. Microb Pathog (2013) 56:21–8 10.1016/j.micpath.2012.10.002 23092690

[81] Hsu SH Lo YY Tung JY Ko YC Sun YJ Hung CC Leptospiral outer membrane lipoprotein LipL32 binding on toll-like receptor 2 of renal cells as determined with an atomic force microscope. Biochemistry (2010) 49(26):5408–17 10.1021/bi100058w 20513152

[82] Grassmann AA Felix SR dos Santos CX Amaral MG Seixas Neto AC Fagundes MQ Protection against lethal leptospirosis after vaccination with LipL32 coupled or coadministered with the B subunit of *Escherichia coli* heat-labile enterotoxin. Clin Vaccine Immunol (2012) 19(5):740–5 10.1128/CVI.05720-11 22379066 PMC3346321

[83] Luo D Xue F Ojcius DM Zhao J Mao Y Li L Protein typing of major outer membrane lipoproteins from Chinese pathogenic *Leptospira* spp. and characterization of their immunogenicity. Vaccine (2009) 28(1):243–55 10.1016/j.vaccine.2009.09.089 19796723

[84] Cullen PA Haake DA Adler B Outer membrane proteins of pathogenic spirochetes. FEMS Microbiol Rev (2004) 28(3):291–318 10.1016/j.femsre.2003.10.004 15449605 PMC2666356

[85] Radolf JD Norgard MV Schulz WW Outer membrane ultrastructure explains the limited antigenicity of virulent *Treponema pallidum*. Proc Natl Acad Sci U S A (1989) 86(6):2051–5 10.1073/pnas.86.6.2051 2648388 PMC286845

[86] Jones JD Bourell KW Norgard MV Radolf JD Membrane topology of *Borrelia burgdorferi* and *Treponema pallidum* lipoproteins. Infect Immun (1995) 63(7):2424–34 7790053 10.1128/iai.63.7.2424-2434.1995 PMC173324

[87] Sellati TJ Bouis DA Caimano MJ Feulner JA Ayers C Lien E Activation of human monocytic cells by *Borrelia burgdorferi* and *Treponema pallidum* is facilitated by CD14 and correlates with surface exposure of spirochetal lipoproteins. J Immunol (1999) 163(4):2049–56 10438943

[88] Underhill DM Ozinsky A Hajjar AM Stevens A Wilson CB Bassetti M The toll-like receptor 2 is recruited to macrophage phagosomes and discriminates between pathogens. Nature (1999) 401(6755):811–5 10.1038/44605 10548109

[89] Biesert L Scheuer W Bessler WG Interaction of mitogenic bacterial lipoprotein and a synthetic analogue with mouse lymphocytes. Isolation and characterization of binding proteins. Eur J Biochem (1987) 162(3):651–7 10.1111/j.1432-1033.1987.tb10687.x 3549292

[90] Hauschildt S Hoffmann P Beuscher HU Dufhues G Heinrich P Wiesmuller KH Activation of bone marrow-derived mouse macrophages by bacterial lipopeptide: cytokine production, phagocytosis and Ia expression. Eur J Immunol (1990) 20(1):63–8 10.1002/eji.1830200110 2407539

[91] Bessler WG Ottenbreit BP Studies on the mitogenic principle of the lipoprotein from the outer membrane of *Escherichia coli*. Biochem Biophys Res Commun (1976) 76(2):239–46 10.1016/0006-291X(77)90717-3 800336

[92] Bessler WG Cox M Lex A Suhr B Wiesmuller KH Jung G Synthetic lipopeptide analogs of bacterial lipoprotein are potent polyclonal activators for murine B lymphocytes. J Immunol (1985) 135(3):1900–5 3874908

[93] Hoffmann P Heinle S Schade UF Loppnow H Ulmer AJ Flad HD Stimulation of human and murine adherent cells by bacterial lipoprotein and synthetic lipopeptide analogues. Immunobiology (1988) 177(2):158–70 10.1016/S0171-2985(88)80036-6 3042614

[94] Norgard MV Riley BS Richardson JA Radolf JD Dermal inflammation elicited by synthetic analogs of *Treponema pallidum* and *Borrelia burgdorferi* lipoproteins. Infect Immun (1995) 63(4):1507–15 7890417 10.1128/iai.63.4.1507-1515.1995 PMC173182

[95] Sellati TJ Abrescia LD Radolf JD Furie MB Outer surface lipoproteins of *Borrelia burgdorferi* activate vascular endothelium *in vitro*. Infect Immun (1996) 64(8):3180–7 8757851 10.1128/iai.64.8.3180-3187.1996 PMC174205

[96] Takeuchi O Sato S Horiuchi T Hoshino K Takeda K Dong Z Cutting edge: role of toll-like receptor 1 in mediating immune response to microbial lipoproteins. J Immunol (2002) 169(1):10–4 10.4049/jimmunol.169.1.10 12077222

[97] Wooten RM Morrison TB Weis JH Wright SD Thieringer R Weis JJ The role of CD14 in signaling mediated by outer membrane lipoproteins of *Borrelia burgdorferi*. J Immunol (1998) 160(11):5485–92 9605151

[98] Zhang JR Hardham JM Barbour AG Norris SJ Antigenic variation in Lyme disease borreliae by promiscuous recombination of VMP-like sequence cassettes. Cell (1997) 89(2):275–85 10.1016/S0092-8674(00)80206-8 9108482

[99] Hellwage J Meri T Heikkila T Alitalo A Panelius J Lahdenne P The complement regulator factor H binds to the surface protein OspE of *Borrelia burgdorferi*. J Biol Chem (2001) 276(11):8427–35 10.1074/jbc.M007994200 11113124

[100] Guo BP Brown EL Dorward DW Rosenberg LC Hook M Decorin-binding adhesins from *Borrelia burgdorferi*. Mol Microbiol (1998) 30(4):711–23 10.1046/j.1365-2958.1998.01103.x 10094620

[101] Fikrig E Feng W Barthold SW Telford SR III Flavell RA Arthropod- and host-specific *Borrelia burgdorferi* bbk32 expression and the inhibition of spirochete transmission. J Immunol (2000) 164(10):5344–51 10.4049/jimmunol.164.10.5344 10799897

[102] Probert WS Johnson BJ Identification of a 47 kDa fibronectin-binding protein expressed by *Borrelia burgdorferi* isolate B31. Mol Microbiol (1998) 30(5):1003–15 10.1046/j.1365-2958.1998.01127.x 9988477

[103] Hauk P Macedo F Romero EC Vasconcellos SA de Morais ZM Barbosa AS In LipL32, the major leptospiral lipoprotein, the C terminus is the primary immunogenic domain and mediates interaction with collagen IV and plasma fibronectin. Infect Immun (2008) 76(6):2642–50 10.1128/IAI.01639-07 18391007 PMC2423089

[104] Choy HA Kelley MM Chen TL Moller AK Matsunaga J Haake DA Physiological osmotic induction of *Leptospira interrogans* adhesion: LigA and LigB bind extracellular matrix proteins and fibrinogen. Infect Immun (2007) 75(5):2441–50 10.1128/IAI.01635-06 17296754 PMC1865782

[105] Pal U de Silva AM Montgomery RR Fish D Anguita J Anderson JF Attachment of *Borrelia burgdorferi* within *Ixodes scapularis* mediated by outer surface protein A. J Clin Invest (2000) 106(4):561–9 10.1172/JCI9427 10953031 PMC380253

[106] Pal U Montgomery RR Lusitani D Voet P Weynants V Malawista SE Inhibition of *Borrelia burgdorferi*-tick interactions *in vivo* by outer surface protein A antibody. J Immunol (2001) 166(12):7398–403 10.4049/jimmunol.166.12.7398 11390491

[107] Stewart PE Hoff J Fischer E Krum JG Rosa PA Genome-wide transposon mutagenesis of *Borrelia burgdorferi* for identification of phenotypic mutants. Appl Environ Microbiol (2004) 70(10):5973–9 10.1128/AEM.70.10.5973-5979.2004 15466540 PMC522107

[108] Deka RK Brautigam CA Biddy BA Liu WZ Norgard MV Evidence for an ABC-type riboflavin transporter system in pathogenic spirochetes. MBio (2013) 4(1):e615–612 10.1128/mBio.00615-12 23404400 PMC3573665

[109] Salazar JC Pope CD Sellati TJ Feder HM Jr Kiely TG Dardick KR Coevolution of markers of innate and adaptive immunity in skin and peripheral blood of patients with erythema migrans. J Immunol (2003) 171(5):2660–70 10.4049/jimmunol.171.9.4934-a 12928420

[110] Erbelding E 2001 Syphilis rates show increase: does this portend a new wave of HIV infection? Hopkins HIV Rep (2003) 15(1):15 12542008

[111] Krutzik SR Ochoa MT Sieling PA Uematsu S Ng YW Legaspi A Activation and regulation of toll-like receptors 2 and 1 in human leprosy. Nat Med (2003) 9(5):525–32 10.1038/nm864 12692544

[112] Schwan TG Piesman J Golde WT Dolan MC Rosa PA Induction of an outer surface protein on *Borrelia burgdorferi* during tick feeding. Proc Natl Acad Sci U S A (1995) 92(7):2909–13 10.1073/pnas.92.7.2909 7708747 PMC42328

[113] Lengl-Janssen B Strauss AF Steere AC Kamradt T The T helper cell response in Lyme arthritis: differential recognition of *Borrelia burgdorferi* outer surface protein A in patients with treatment-resistant or treatment-responsive Lyme arthritis. J Exp Med (1994) 180(6):2069–78 10.1084/jem.180.6.2069 7964484 PMC2191805

[114] de Silva AM Fikrig E Arthropod- and host-specific gene expression by *Borrelia burgdorferi*. J Clin Invest (1997) 99(3):377–9 10.1172/JCI119169 9022068 PMC507808

[115] Wooten RM Modur VR McIntyre TM Weis JJ *Borrelia burgdorferi* outer membrane protein A induces nuclear translocation of nuclear factor-kappa B and inflammatory activation in human endothelial cells. J Immunol (1996) 157(10):4584–90 8906837

[116] Morrison TB Weis JH Weis JJ *Borrelia burgdorferi* outer surface protein A (OspA) activates and primes human neutrophils. J Immunol (1997) 158(10):4838–45 9144499

[117] Bunikis J Barbour AG Access of antibody or trypsin to an integral outer membrane protein (P66) of *Borrelia burgdorferi* is hindered by Osp lipoproteins. Infect Immun (1999) 67(6):2874–83 10338494 10.1128/iai.67.6.2874-2883.1999 PMC96595

[118] Hinnebusch BJ Barbour AG Restrepo BI Schwan TG Population structure of the relapsing fever spirochete *Borrelia hermsii* as indicated by polymorphism of two multigene families that encode immunogenic outer surface lipoproteins. Infect Immun (1998) 66(2):432–40 9453591 10.1128/iai.66.2.432-440.1998 PMC107923

[119] Zhang JR Norris SJ Genetic variation of the *Borrelia burgdorferi* gene vlsE involves cassette-specific, segmental gene conversion. Infect Immun (1998) 66(8):3698–704 9673251 10.1128/iai.66.8.3698-3704.1998 PMC108404

[120] Zhang JR Norris SJ Kinetics and *in vivo* induction of genetic variation of vlsE in *Borrelia burgdorferi*. Infect Immun (1998) 66(8):3689–97 9673250 10.1128/iai.66.8.3689-3697.1998 PMC108403

[121] Rogovskyy AS Bankhead T Variable VlsE is critical for host reinfection by the Lyme disease spirochete. PLoS One (2013) 8(4):e61226 10.1371/journal.pone.0061226 23593438 PMC3620393

[122] Vidal V Cutler S Scragg IG Wright DJ Kwiatkowski D Characterisation of silent and active genes for a variable large protein of *Borrelia recurrentis*. BMC Infect Dis (2002) 2:25 10.1186/1471-2334-2-25 12377101 PMC130189

[123] Schwan TG Hinnebusch BJ Bloodstream- versus tick-associated variants of a relapsing fever bacterium. Science (1998) 280(5371):1938–40 10.1126/science.280.5371.1938 9632392

[124] Carter CJ Bergstrom S Norris SJ Barbour AG A family of surface-exposed proteins of 20 kilodaltons in the genus *Borrelia*. Infect Immun (1994) 62(7):2792–9 8005669 10.1128/iai.62.7.2792-2799.1994 PMC302883

[125] Bolz DD Sundsbak RS Ma Y Akira S Kirschning CJ Zachary JF MyD88 plays a unique role in host defense but not arthritis development in Lyme disease. J Immunol (2004) 173(3):2003–10 10.4049/jimmunol.173.3.2003 15265935

[126] Benhnia MR Wroblewski D Akhtar MN Patel RA Lavezzi W Gangloff SC Signaling through CD14 attenuates the inflammatory response to *Borrelia burgdorferi*, the agent of Lyme disease. J Immunol (2005) 174(3):1539–48 10.4049/jimmunol.174.3.1539 15661914

[127] Wang G Ma Y Buyuk A McClain S Weis JJ Schwartz I Impaired host defense to infection and toll-like receptor 2-independent killing of *Borrelia burgdorferi* clinical isolates in TLR2-deficient C3H/HeJ mice. FEMS Microbiol Lett (2004) 231(2):219–25 10.1016/S0378-1097(03)00960-1 14987768

[128] Wooten RM Ma Y Yoder RA Brown JP Weis JH Zachary JF Toll-like receptor 2 is required for innate, but not acquired, host defense to *Borrelia burgdorferi*. J Immunol (2002) 168(1):348–55 10.4049/jimmunol.168.1.348 11751980

[129] Wooten RM Weis JJ Host-pathogen interactions promoting inflammatory Lyme arthritis: use of mouse models for dissection of disease processes. Curr Opin Microbiol (2001) 4(3):274–9 10.1016/S1369-5274(00)00202-2 11378478

[130] Schroder NW Diterich I Zinke A Eckert J Draing C von Baehr V Heterozygous Arg753Gln polymorphism of human TLR-2 impairs immune activation by *Borrelia burgdorferi* and protects from late stage Lyme disease. J Immunol (2005) 175(4):2534–40 10.4049/jimmunol.175.4.2534 16081826

[131] Bas S James RW Gabay C Serum lipoproteins attenuate macrophage activation and toll-like receptor stimulation by bacterial lipoproteins. BMC Immunol (2010) 11:46 10.1186/1471-2172-11-46 20846396 PMC2949775

[132] Alitalo A Meri T Comstedt P Jeffery L Tornberg J Strandin T Expression of complement factor H binding immunoevasion proteins in *Borrelia garinii* isolated from patients with neuroborreliosis. Eur J Immunol (2005) 35(10):3043–53 10.1002/eji.200526354 16208765

[133] Alitalo A Meri T Ramo L Jokiranta TS Heikkila T Seppala IJ Complement evasion by *Borrelia burgdorferi*: serum-resistant strains promote C3b inactivation. Infect Immun (2001) 69(6):3685–91 10.1128/IAI.69.6.3685-3691.2001 11349031 PMC98369

[134] Skerka C Zipfel PF Complement factor H related proteins in immune diseases. Vaccine (2008) 26(Suppl 8):I9–14 10.1016/j.vaccine.2008.11.021 19388158

[135] Kraiczy P Skerka C Brade V Zipfel PF Further characterization of complement regulator-acquiring surface proteins of *Borrelia burgdorferi*. Infect Immun (2001) 69(12):7800–9 10.1128/IAI.69.12.7800-7809.2001 11705962 PMC98876

[136] Bhattacharjee A Oeemig JS Kolodziejczyk R Meri T Kajander T Lehtinen MJ Structural basis for complement evasion by Lyme disease pathogen *Borrelia burgdorferi*. J Biol Chem (2013) 288(26):18685–95 10.1074/jbc.M113.459040 23658013 PMC3696643

[137] Stevenson B El-Hage N Hines MA Miller JC Babb K Differential binding of host complement inhibitor factor H by *Borrelia burgdorferi* Erp surface proteins: a possible mechanism underlying the expansive host range of Lyme disease spirochetes. Infect Immun (2002) 70(2):491–7 10.1128/IAI.70.2.491-497.2002 11796574 PMC127719

[138] Matsunaga J Schlax PJ Haake DA Role for cis-acting RNA sequences in the temperature-dependent expression of the multiadhesive lig proteins in *Leptospira interrogans*. J Bacteriol (2013) 195(22):5092–101 10.1128/JB.00663-13 24013626 PMC3811586

[139] Nowling JM Philipp MT Killing of *Borrelia burgdorferi* by antibody elicited by OspA vaccine is inefficient in the absence of complement. Infect Immun (1999) 67(1):443–5 9864253 10.1128/iai.67.1.443-445.1999 PMC96334

[140] Kenedy MR Akins DR The OspE-related proteins inhibit complement deposition and enhance serum resistance of *Borrelia burgdorferi*, the lyme disease spirochete. Infect Immun (2011) 79(4):1451–7 10.1128/IAI.01274-10 21282413 PMC3067540

[141] Kenedy MR Vuppala SR Siegel C Kraiczy P Akins DR CspA-mediated binding of human factor H inhibits complement deposition and confers serum resistance in *Borrelia burgdorferi*. Infect Immun (2009) 77(7):2773–82 10.1128/IAI.00318-09 19451251 PMC2708555

[142] Hartmann K Corvey C Skerka C Kirschfink M Karas M Brade V Functional characterization of BbCRASP-2, a distinct outer membrane protein of *Borrelia burgdorferi* that binds host complement regulators factor H and FHL-1. Mol Microbiol (2006) 61(5):1220–36 10.1111/j.1365-2958.2006.05318.x 16925556

[143] Panelius J Meri T Seppala I Eholuoto M Alitalo A Meri S Outer surface protein E antibody response and its effect on complement factor H binding to OspE in Lyme borreliosis. Microbes Infect (2008) 10(2):135–42 10.1016/j.micinf.2007.10.016 18248762

[144] Kraiczy P Hartmann K Hellwage J Skerka C Kirschfink M Brade V Immunological characterization of the complement regulator factor H-binding CRASP and Erp proteins of *Borrelia burgdorferi*. Int J Med Microbiol (2004) 293(Suppl 37):152–7 10.1016/S1433-1128(04)80029-9 15146998

[145] Brooks CS Vuppala SR Jett AM Alitalo A Meri S Akins DR Complement regulator-acquiring surface protein 1 imparts resistance to human serum in *Borrelia burgdorferi*. J Immunol (2005) 175(5):3299–308 10.4049/jimmunol.175.5.3299 16116222

[146] Coleman AS Yang X Kumar M Zhang X Promnares K Shroder D *Borrelia burgdorferi* complement regulator-acquiring surface protein 2 does not contribute to complement resistance or host infectivity. PLoS One (2008) 3(8):3010e 10.1371/journal.pone.0003010 18714378 PMC2526170

[147] Bykowski T Woodman ME Cooley AE Brissette CA Wallich R Brade V *Borrelia burgdorferi* complement regulator-acquiring surface proteins (BbCRASPs): expression patterns during the mammal-tick infection cycle. Int J Med Microbiol (2008) 298(Suppl 1):249–56 10.1016/j.ijmm.2007.10.002 18165150 PMC2551708

[148] Benach JL Coleman JL Garcia-Monco JC Deponte PC Biological activity of *Borrelia burgdorferi* antigens. Ann N Y Acad Sci (1988) 539:115–25 10.1111/j.1749-6632.1988.tb31845.x 2461134

[149] Cinco M Panfili E Presani G Perticarari S Interaction with *Borrelia burgdorferi* causes increased expression of the CR3 integrin and increased binding affinity to fibronectin via CR3. J Mol Microbiol Biotechnol (2000) 2(4):575–9 11075934

[150] Garcia RC Murgia R Cinco M Complement receptor 3 binds the *Borrelia burgdorferi* outer surface proteins OspA and OspB in an iC3b-independent manner. Infect Immun (2005) 73(9):6138–42 10.1128/IAI.73.9.6138-6142.2005 16113335 PMC1231105

[151] Wright SD Ramos RA Tobias PS Ulevitch RJ Mathison JC CD14, a receptor for complexes of lipopolysaccharide (LPS) and LPS binding protein. Science (1990) 249(4975):1431–3 10.1126/science.1698311 1698311

[152] Ranoa DR Kelley SL Tapping RI Human lipopolysaccharide-binding protein (LBP) and CD14 independently deliver triacylated lipoproteins to toll-like receptor 1 (TLR1) and TLR2 and enhance formation of the ternary signaling complex. J Biol Chem (2013) 288(14):9729–41 10.1074/jbc.M113.453266 23430250 PMC3617275

[153] Marre ML Petnicki-Ocwieja T DeFrancesco AS Darcy CT Hu LT Human integrin alpha(3)beta(1) regulates TLR2 recognition of lipopeptides from endosomal compartments. PLoS One (2010) 5(9):e12871 10.1371/journal.pone.0012871 20877569 PMC2943923

[154] Behera AK Durand E Cugini C Antonara S Bourassa L Hildebrand E *Borrelia burgdorferi* BBB07 interaction with integrin alpha3beta1 stimulates production of pro-inflammatory mediators in primary human chondrocytes. Cell Microbiol (2008) 10(2):320–31 10.1111/j.1462-5822.2007.01043.x 17822440 PMC2586958

[155] Coburn J Cugini C Targeted mutation of the outer membrane protein P66 disrupts attachment of the Lyme disease agent, *Borrelia burgdorferi*, to integrin alphavbeta3. Proc Natl Acad Sci U S A (2003) 100(12):7301–6 10.1073/pnas.1131117100 12748384 PMC165870

[156] Talkington J Nickell SP Role of Fc gamma receptors in triggering host cell activation and cytokine release by *Borrelia burgdorferi*. Infect Immun (2001) 69(1):413–9 10.1128/IAI.69.1.413-419.2001 11119532 PMC97898

[157] Haupl T Landgraf S Netusil P Biller N Capiau C Desmons P Activation of monocytes by three OspA vaccine candidates: lipoprotein OspA is a potent stimulator of monokines. FEMS Immunol Med Microbiol (1997) 19(1):15–23 10.1016/S0928-8244(97)00046-1 9322065

[158] Murthy PK Dennis VA Lasater BL Philipp MT Interleukin-10 modulates proinflammatory cytokines in the human monocytic cell line THP-1 stimulated with *Borrelia burgdorferi* lipoproteins. Infect Immun (2000) 68(12):6663–9 10.1128/IAI.68.12.6663-6669.2000 11083779 PMC97764

[159] Gautam A Dixit S Philipp MT Singh SR Morici LA Kaushal D Interleukin-10 alters effector functions of multiple genes induced by *Borrelia burgdorferi* in macrophages to regulate Lyme disease inflammation. Infect Immun (2011) 79(12):4876–92 10.1128/IAI.05451-11 21947773 PMC3232652

[160] Gautam A Dixit S Embers M Gautam R Philipp MT Singh SR Different patterns of expression and of IL-10 modulation of inflammatory mediators from macrophages of Lyme disease-resistant and -susceptible mice. PLoS One (2012) 7(9):e43860 10.1371/journal.pone.0043860 23024745 PMC3443101

[161] Ganapamo F Dennis VA Philipp MT Differential acquired immune responsiveness to bacterial lipoproteins in Lyme disease-resistant and -susceptible mouse strains. Eur J Immunol (2003) 33(7):1934–40 10.1002/eji.200323655 12884859

[162] van Seventer GA Shimizu Y Horgan KJ Shaw S The LFA-1 ligand ICAM-1 provides an important costimulatory signal for T cell receptor-mediated activation of resting T cells. J Immunol (1990) 144(12):4579–86 1972160

[163] Bouis DA Popova TG Takashima A Norgard MV Dendritic cells phagocytose and are activated by *Treponema pallidum*. Infect Immun (2001) 69(1):518–28 10.1128/IAI.69.1.518-528.2001 11119545 PMC97911

[164] Gondolf KB Mihatsch M Curschellas E Dunn JJ Batsford SR Induction of experimental allergic arthritis with outer surface proteins of *Borrelia burgdorferi*. Arthritis Rheum (1994) 37(7):1070–7 10.1002/art.1780370713 8024615

[165] Guerrier G D’Ortenzio E The Jarisch-Herxheimerreaction in leptospirosis: a systematic review. PLoS One (2013) 8(3):e59266 10.1371/journal.pone.0059266 23555644 PMC3608636

[166] Belum GR Belum VR Chaitanya Arudra SK Reddy BS The Jarisch-Herxheimer reaction: revisited. Travel Med Infect Dis (2013) 11(4):231–7 10.1016/j.tmaid.2013.04.001 23632012

[167] Kupper TS Fuhlbrigge RC Immune surveillance in the skin: mechanisms and clinical consequences. Nat Rev Immunol (2004) 4(3):211–22 10.1038/nri1310 15039758 PMC7097017

[168] Gergel EI Furie MB Populations of human T lymphocytes that traverse the vascular endothelium stimulated by *Borrelia burgdorferi* are enriched with cells that secrete gamma interferon. Infect Immun (2004) 72(3):1530–6 10.1128/IAI.72.3.1530-1536.2004 14977959 PMC356023

[169] Pivarcsi A Bodai L Rethi B Kenderessy-Szabo A Koreck A Szell M Expression and function of toll-like receptors 2 and 4 in human keratinocytes. Int Immunol (2003) 15(6):721–30 10.1093/intimm/dxg068 12750356

[170] Takeuchi J Watari E Shinya E Norose Y Matsumoto M Seya T Down-regulation of toll-like receptor expression in monocyte-derived Langerhans cell-like cells: implications of low-responsiveness to bacterial components in the epidermal Langerhans cells. Biochem Biophys Res Commun (2003) 306(3):674–9 10.1016/S0006-291X(03)01022-2 12810071

[171] Cadavid D Thomas DD Crawley R Barbour AG Variability of a bacterial surface protein and disease expression in a possible mouse model of systemic Lyme borreliosis. J Exp Med (1994) 179(2):631–42 10.1084/jem.179.2.631 8294872 PMC2191368

[172] Rawadi G Garcia J Lemercier B Roman-Roman S Signal transduction pathways involved in the activation of NF-kappa B, AP-1, and c-fos by *Mycoplasma fermentans* membrane lipoproteins in macrophages. J Immunol (1999) 162(4):2193–203 9973495

[173] Zhang H Peterson JW Niesel DW Klimpel GR Bacterial lipoprotein and lipopolysaccharide act synergistically to induce lethal shock and proinflammatory cytokine production. J Immunol (1997) 159(10):4868–78 9366412

[174] Blanco DR Reimann K Skare J Champion CI Foley D Exner MM Isolation of the outer membranes from *Treponema pallidum* and *Treponema vincentii*. J Bacteriol (1994) 176(19):6088–99 7928971 10.1128/jb.176.19.6088-6099.1994 PMC196829

[175] Radolf JD Robinson EJ Bourell KW Akins DR Porcella SF Weigel LM Characterization of outer membranes isolated from *Treponema pallidum*, the syphilis spirochete. Infect Immun (1995) 63(11):4244–52 7591054 10.1128/iai.63.11.4244-4252.1995 PMC173603

[176] Radolf JD *Treponema pallidum* and the quest for outer membrane proteins. Mol Microbiol (1995) 16(6):1067–73 10.1111/j.1365-2958.1995.tb02332.x 8577243

[177] Cameron CE Castro C Lukehart SA Van Voorhis WC Function and protective capacity of *Treponema pallidum* subsp. pallidum glycerophosphodiester phosphodiesterase. Infect Immun (1998) 66(12):5763–70 9826352 10.1128/iai.66.12.5763-5770.1998 PMC108728

[178] Miller DP McDowell JV Bell JK Marconi RT Crystallization of the factor H-binding protein, FhbB, from the periopathogen *Treponema denticola*. Acta Crystallogr Sect F Struct Biol Cryst Commun (2011) 67(Pt 6):678–81 10.1107/S1744309111011298 21636910 PMC3107141

[179] Miller DP Bell JK McDowell JV Conrad DH Burgner JW Heroux A Structure of factor H-binding protein B (FhbB) of the periopathogen, *Treponema denticola*: insights into progression of periodontal disease. J Biol Chem (2012) 287(16):12715–22 10.1074/jbc.M112.339721 22371503 PMC3339992

[180] Miller DP McDowell JV Rhodes DV Allard A Caimano M Bell JK Sequence divergence in the *Treponema denticola* FhbB protein and its impact on factor H binding. Mol Oral Microbiol (2013) 28(4):316–30 10.1111/omi.12027 23601078 PMC3785937

[181] McDowell JV Lankford J Stamm L Sadlon T Gordon DL Marconi RT Demonstration of factor H-like protein 1 binding to *Treponema denticola*, a pathogen associated with periodontal disease in humans. Infect Immun (2005) 73(11):7126–32 10.1128/IAI.73.11.7126-7132.2005 16239506 PMC1273895

[182] McDowell JV Frederick J Stamm L Marconi RT Identification of the gene encoding the FhbB protein of *Treponema denticola*, a highly unique factor H-like protein 1 binding protein. Infect Immun (2007) 75(2):1050–4 10.1128/IAI.01458-06 17101650 PMC1828522

[183] McDowell JV Huang B Fenno JC Marconi RT Analysis of a unique interaction between the complement regulatory protein factor H and the periodontal pathogen *Treponema denticola*. Infect Immun (2009) 77(4):1417–25 10.1128/IAI.01544-08 19204088 PMC2663137

[184] McDowell JV Frederick J Miller DP Goetting-Minesky MP Goodman H Fenno JC Identification of the primary mechanism of complement evasion by the periodontal pathogen, *Treponema denticola*. Mol Oral Microbiol (2011) 26(2):140–9 10.1111/j.2041-1014.2010.00598.x 21375704 PMC3053026

[185] Rosen G Sela MN Naor R Halabi A Barak V Shapira L Activation of murine macrophages by lipoprotein and lipooligosaccharide of *Treponema denticola*. Infect Immun (1999) 67(3):1180–6 10024558 10.1128/iai.67.3.1180-1186.1999 PMC96444

[186] Sela MN Bolotin A Naor R Weinberg A Rosen G Lipoproteins of *Treponema denticola*: their effect on human polymorphonuclear neutrophils. J Periodontal Res (1997) 32(5):455–66 10.1111/j.1600-0765.1997.tb00558.x 9266497

[187] Veith PD Dashper SG O’Brien-Simpson NM Paolini RA Orth R Walsh KA Major proteins and antigens of *Treponema denticola*. Biochim Biophys Acta (2009) 1794(10):1421–32 10.1016/j.bbapap.2009.06.001 19501677

[188] Ishihara K Virulence factors of *Treponema denticola*. Periodontol 2000 (2010) 54(1):117–35 10.1111/j.1600-0757.2009.00345.x 20712637

[189] Li X Strle K Wang P Acosta DI McHugh GA Sikand N Tick-specific borrelial antigens appear to be upregulated in American but not European patients with Lyme arthritis, a late manifestation of Lyme borreliosis. J Infect Dis (2013) 208(6):934–41 10.1093/infdis/jit269 23766526 PMC3749008

[190] Strle K Shin JJ Glickstein LJ Steere AC Association of a toll-like receptor 1 polymorphism with heightened Th1 inflammatory responses and antibiotic-refractory Lyme arthritis. Arthritis Rheum (2012) 64(5):1497–507 10.1002/art.34383 22246581 PMC3338893

[191] Steere AC Klitz W Drouin EE Falk BA Kwok WW Nepom GT Antibiotic-refractory Lyme arthritis is associated with HLA-DR molecules that bind a *Borrelia burgdorferi* peptide. J Exp Med (2006) 203(4):961–71 10.1084/jem.20052471 16585267 PMC3212725

[192] Bankhead T Chaconas G The role of VlsE antigenic variation in the Lyme disease spirochete: persistence through a mechanism that differs from other pathogens. Mol Microbiol (2007) 65(6):1547–58 10.1111/j.1365-2958.2007.05895.x 17714442

[193] Philipp MT Bowers LC Fawcett PT Jacobs MB Liang FT Marques AR Antibody response to IR6, a conserved immunodominant region of the VlsE lipoprotein, wanes rapidly after antibiotic treatment of *Borrelia burgdorferi* infection in experimental animals and in humans. J Infect Dis (2001) 184(7):870–8 10.1086/323392 11550127

[194] de Silva AM Zeidner NS Zhang Y Dolan MC Piesman J Fikrig E Influence of outer surface protein A antibody on *Borrelia burgdorferi* within feeding ticks. Infect Immun (1999) 67(1):30–5 9864192 10.1128/iai.67.1.30-35.1999 PMC96273

[195] Erdile LF Brandt MA Warakomski DJ Westrack GJ Sadziene A Barbour AG Role of attached lipid in immunogenicity of *Borrelia burgdorferi* OspA. Infect Immun (1993) 61(1):81–90 8418068 10.1128/iai.61.1.81-90.1993 PMC302690

[196] Sears JE Fikrig E Nakagawa TY Deponte K Marcantonio N Kantor FS Molecular mapping of Osp-A mediated immunity against *Borrelia burgdorferi*, the agent of Lyme disease. J Immunol (1991) 147(6):1995–2000 1716290

[197] Johnson BJ Sviat SL Happ CM Dunn JJ Frantz JC Mayer LW Incomplete protection of hamsters vaccinated with unlipidated OspA from *Borrelia burgdorferi* infection is associated with low levels of antibody to an epitope defined by mAb LA-2. Vaccine (1995) 13(12):1086–94 10.1016/0264-410X(95)00035-Y 7491816

[198] Hanson MS Cassatt DR Guo BP Patel NK McCarthy MP Dorward DW Active and passive immunity against *Borrelia burgdorferi* decorin binding protein A (DbpA) protects against infection. Infect Immun (1998) 66(5):2143–53 9573101 10.1128/iai.66.5.2143-2153.1998 PMC108175

[199] Zuckert WR A call to order at the spirochaetal host-pathogen interface. Mol Microbiol (2013) 89(2):207–11 10.1111/mmi.12286 23750784 PMC3746072

[200] Norman MU Moriarty TJ Dresser AR Millen B Kubes P Chaconas G Molecular mechanisms involved in vascular interactions of the Lyme disease pathogen in a living host. PLoS Pathog (2008) 4(10):e1000169 10.1371/journal.ppat.1000169 18833295 PMC2542414

[201] Yang X Qin J Promnares K Kariu T Anderson JF Pal U Novel microbial virulence factor triggers murine lyme arthritis. J Infect Dis (2013) 207(6):907–18 10.1093/infdis/jis930 23303811 PMC3571445

[202] Vidal V Scragg IG Cutler SJ Rockett KA Fekade D Warrell DA Variable major lipoprotein is a principal TNF-inducing factor of louse-borne relapsing fever. Nat Med (1998) 4(12):1416–20 10.1038/4007 9846580

[203] Rossmann E Kraiczy P Herzberger P Skerka C Kirschfink M Simon MM Dual binding specificity of a *Borrelia hermsii*-associated complement regulator-acquiring surface protein for factor H and plasminogen discloses a putative virulence factor of relapsing fever spirochetes. J Immunol (2007) 178(11):7292–301 10.4049/jimmunol.178.11.7292 17513779

[204] Grosskinsky S Schott M Brenner C Cutler SJ Kraiczy P Zipfel PF *Borrelia recurrentis* employs a novel multifunctional surface protein with anti-complement, anti-opsonic and invasive potential to escape innate immunity. PLoS One (2009) 4(3):e4858 10.1371/journal.pone.0004858 19308255 PMC2654920

[205] Hovis KM Jones JP Sadlon T Raval G Gordon DL Marconi RT Molecular analyses of the interaction of *Borrelia hermsii* FhbA with the complement regulatory proteins factor H and factor H-like protein 1. Infect Immun (2006) 74(4):2007–14 10.1128/IAI.74.4.2007-2014.2006 16552029 PMC1418896

[206] Hovis KM McDowell JV Griffin L Marconi RT Identification and characterization of a linear-plasmid-encoded factor H-binding protein (FhbA) of the relapsing fever spirochete *Borrelia hermsii*. J Bacteriol (2004) 186(9):2612–8 10.1128/JB.186.9.2612-2618.2004 15090501 PMC387808

[207] Grosskinsky S Schott M Brenner C Cutler SJ Simon MM Wallich R Human complement regulators C4b-binding protein and C1 esterase inhibitor interact with a novel outer surface protein of *Borrelia recurrentis*. PLoS Negl Trop Dis (2010) 4(6):e698 10.1371/journal.pntd.0000698 20532227 PMC2879370

[208] Werts C Tapping RI Mathison JC Chuang TH Kravchenko V Saint G I Leptospiral lipopolysaccharide activates cells through a TLR2-dependent mechanism. Nat Immunol (2001) 2(4):346–52 10.1038/86354 11276206

[209] Haake DA Mazel MK McCoy AM Milward F Chao G Matsunaga J Leptospiral outer membrane proteins OmpL1 and LipL41 exhibit synergistic immunoprotection. Infect Immun (1999) 67(12):6572–82 10569777 10.1128/iai.67.12.6572-6582.1999 PMC97069

[210] Verma A Artiushin S Matsunaga J Haake DA Timoney JF LruA and LruB, novel lipoproteins of pathogenic *Leptospira interrogans* associated with equine recurrent uveitis. Infect Immun (2005) 73(11):7259–66 10.1128/IAI.73.11.7259-7266.2005 16239521 PMC1273856

[211] Verma A Rathinam SR Priya CG Muthukkaruppan VR Stevenson B Timoney JF LruA and LruB antibodies in sera of humans with leptospiral uveitis. Clin Vaccine Immunol (2008) 15(6):1019–23 10.1128/CVI.00203-07 18400972 PMC2446624

[212] Verma A Kumar P Babb K Timoney JF Stevenson B Cross-reactivity of antibodies against leptospiral recurrent uveitis-associated proteins A and B (LruA and LruB) with eye proteins. PLoS Negl Trop Dis (2010) 4(8):e778 10.1371/journal.pntd.0000778 20689825 PMC2914785

[213] Zhang K Murray GL Seemann T Srikram A Bartpho T Sermswan RW Leptospiral LruA is required for virulence and modulates an interaction with mammalian apolipoprotein AI. Infect Immun (2013) 81(10):3872–9 10.1128/IAI.01195-12 23918777 PMC3811782

[214] Cinco M Domenis R Perticarari S Presani G Marangoni A Blasi E Interaction of leptospires with murine microglial cells. New Microbiol (2006) 29(3):193–9 17058786

[215] Blasi E Ardizzoni A Colombari B Neglia R Baschieri C Peppoloni S NF-kB activation and p38 phosphorylation in microglial cells infected with *Leptospira* or exposed to partially purified leptospiral lipoproteins. Microb Pathog (2007) 42(2–3):80–7 10.1016/j.micpath.2006.11.002 17189679

[216] Klimpel GR Matthias MA Vinetz JM *Leptospira interrogans* activation of human peripheral blood mononuclear cells: preferential expansion of TCR gamma delta+ T cells vs TCR alpha beta+ T cells. J Immunol (2003) 171(3):1447–55 10.4049/jimmunol.171.3.1447 12874237

[217] Nally JE Whitelegge JP Bassilian S Blanco DR Lovett MA Characterization of the outer membrane proteome of *Leptospira interrogans* expressed during acute lethal infection. Infect Immun (2007) 75(2):766–73 10.1128/IAI.00741-06 17101664 PMC1828474

[218] Habarta A Abreu PA Olivera N Hauk P Cedola MT Ferrer MF Increased immunogenicity to LipL32 of *Leptospira interrogans* when expressed as a fusion protein with the cholera toxin B subunit. Curr Microbiol (2011) 62(2):526–31 10.1007/s00284-010-9739-6 20721666

[219] Seixas FK Fernandes CH Hartwig DD Conceicao FR Aleixo JA Dellagostin OA Evaluation of different ways of presenting LipL32 to the immune system with the aim of developing a recombinant vaccine against leptospirosis. Can J Microbiol (2007) 53(4):472–9 10.1139/w06-138 17612601

[220] Silva EF Medeiros MA McBride AJ Matsunaga J Esteves GS Ramos JG The terminal portion of leptospiral immunoglobulin-like protein LigA confers protective immunity against lethal infection in the hamster model of leptospirosis. Vaccine (2007) 25(33):6277–86 10.1016/j.vaccine.2007.05.053 17629368 PMC1994161

[221] Koizumi N Watanabe H Leptospiral immunoglobulin-like proteins elicit protective immunity. Vaccine (2004) 22(11–12):1545–52 10.1016/j.vaccine.2003.10.007 15063580

[222] Cinco M New insights into the pathogenicity of leptospires: evasion of host defences. New Microbiol (2010) 33(4):283–92 21213586

[223] La T Phillips ND Reichel MP Hampson DJ Protection of pigs from swine dysentery by vaccination with recombinant BmpB, a 29.7 kDa outer-membrane lipoprotein of *Brachyspira hyodysenteriae*. Vet Microbiol (2004) 102(1–2):97–109 10.1016/j.vetmic.2004.06.004 15288932

