# Supplementary material for: The Cross-Talk between Spirochetal Lipoproteins and Immunity
Source: Front Immunol. 2014 Jun 30;5:310. doi: 10.3389/fimmu.2014.00310 (PMC4075078; doi:10.3389/fimmu.2014.00310)
Supplement: Supplementary file 1 [file Data_Sheet_1.DOCX]

**Supplemental Table 1. Immunoregulatory effects of other spirochetal lipoproteins.**

| **Bacteria** | **Immune activation** | **Immune evasion** |
| --- | --- | --- |
| ***Treponema pallidum*** | - Major treponemal lipoproteins such as TpN17 (Outer-membrane protein) and TpN47(Zinc-dependent carboxypeptidase) (2;178-180):  1. Contribute to the immunopathogenesis of syphilis *in vivo* (47) 2. Activate directly host vascular endothelium which plays important roles in lymphocyte homing and hemostasis (49) 3. Stimulate macrophage function and production of cytokines such as TNF-a, IL-1 beta, IL-6, and IL-12,(18;44;45;48)  - *T. pallidum* glycerophosphodiester phosphodiesterase (181) has been used as immunogen for vaccine trials |  |
| ***Oral treponemes such as Treponema denticola*** | - Major lipoproteins such as FhbB (factor H-like protein binding protein)(182-188) induce the production of inflammatory mediators (NO, TNF-a, and IL-1) by human macrophages, which in turn may stimulate tissue breakdown as observed in periodontal diseases(189) - An enriched delipidated lipoprotein fraction from *T. denticola* may trigger the production of oxygen radicals and induced lysozyme release from human polymorphonuclear neutrophils(190) - The major TmpC, MglB, Msp, PrcA, OppA and OppA10 lipoproteins were found to be antigenic, consistent with the known high immunogenicity of treponemal lipoproteins (191) | - Factor H-like protein-1 binding by *T. denticola* facilitates adherence to the extracellular matrix in addition to C3b inactivation(192) |
| ***Borrelia Burgdorferi*** | - Inflammatory mediators that are induced by *B. burgdorferi* lipoproteins in cells from mice of Lyme disease-resistant and -susceptible strains have been shown to be tightly regulated by the anti-inflammatory cytokine IL-10(126;130-134). - *B. burgdorferi* lipoproteins such as OspA initiate monocyte activation via different cell surface events but that the signaling pathways ultimately converge to produce qualitatively similar cellular responses (NF-kappa B translocation, CD14-dependent activation pathway) (46;58;59;61) - Antibodies to OspA and BicA have been noted in patients with Lyme arthritis (162;193-195) - DbpA immunization acts by aborting infection after transmission (2) - Integrin α3β1 may co-operate with TLR2/TLR1 in mediating pro-inflammatory responses in human macrophages stimulated with spirochetal lipopeptides such as BBB07 (122;123). - VlsE might be a T-cell independent antigen that could directly stimulate B cells (196;197). - OspA (102;198-201) OspE (99), decorin-binding protein A (DbpA)(202) have been used as immunogens for vaccine trials | - OspC plays a unique role during initial infection while the antigenically variant VlsE proteins allow for persistence in the mammalian host (203) - CspA inhibits complement deposition and enhances serum resistance of *B. burgdorferi* *in vitro* (104) - The OspE and Erp surface proteins of B. burgdorferi recruits complement regulator factor H (FH) onto the bacterial surface to evade complement-mediated cell lysis, an important arm of innate immunity (99;100;103). - The surface lipoprotein, VlsE, alters its antigenic properties and allows the spirochete to evade the host's antibody-mediated response (64;81-83) - BBK32, of *B. burgdorferi* mediates endothelial interactions *in vivo*, thereby facilitating microvascular interactions(204) - BBA57 deficiency reduces the expression of selected neutrophil-recruiting chemokines and associated receptors, causing significant impairment of neutrophil chemotaxis (205). BBA57 is implicated in the pathogenesis of Lyme arthritis and carditis (205). |
| ***Borrelia recurrentis, Borrelia hermsii***  ***Borrelia turicatae*** | - Variable major lipoprotein is a principal TNF-inducing factor of louse-borne relapsing fever that stimulates human monocytes to produce tumor necrosis factor (206) | - Antigenic variation in borrelias may result from recombination of variable large and small protein genes (84) and the diversity of vmp variants allows these pathogens to evade immunity (2) - *B. hermsii* and *B. recurrentis*, specifically bind complement regulatory proteins, i.e. CFH and CFHR-1, via their outer surface lipoproteins FhbA, BhCRASP-1 and HcpA, respectively(207-210) - CihC, binds C4bp and C1-Inh, the major inhibitors of the classical and lectin pathway of complement activation(211) - HcpA binds human complement regulators, Factor H, CFHR-1 (208) |
| ***Borrelia garinii*** | - Surface lipoproteins containing the Pam3C motif induced secretion of CXCL13 from human monocytes (154) |  |
| ***Leptospira interrogans*** | - For intact *L. interrogans*, LPS rather than lipoprotein is the main signaling component for macrophages through a TLR2 pathway (212) - Immunization with a combination of transmembrane porin (OmpL1) and a LipL41 lipoprotein provides synergistic protection and suggests that surface lipoproteins may interfere with the access of antibodies to other spirochetal proteins (213) - Antibody responses to LruA induced during equine and human infection may play an important role in leptospiral uveitis (214-217) - Leptospiral lipoprotein extracts activate p38 phosphorylation and NF-kB and release cytokines and NO in monocytes (218-220) - Loa22 elicits an immune response in human patients (221) - LipL32 can elicit strong immune response when presented to immune system by certain delivery systems, such as Cholera toxin B subunit (222), B subunit of the *Escherichia coli* heat-labile enterotoxin (LTB)(171) or *Mycobacterium bovis* BCG (223) - Lip41 (172;224) and immunoglobulin-like proteins (225;226) have been used as immunogens for vaccine trials | - The putative lipoproteins LigA and LigB promote adhesion of Leptospira to host proteins and capture complement regulators (101) - Len A binds human factor H and FHR-1 and LenB binds HFH (227) - The lipoprotein LIC11207 may modulate the apoptosis of polymorphonuclear cells (PMNs) (54) |
| ***Brachyspira spp.***  ***(B. hyodysenteriae, B. pilosicoli*** | - BmpB appears to have potential as an swine dysentery vaccine component (228) |  |

Abbreviations: BBA57: *Borrelia burgdorferi* A57 protein, BBK32: *Borrelia burgdorferi* 32 kDa fibronectin-binding protein, BhCRASP-1: *Borrelia hermsii* complement regulator-acquiring surface protein 1, C1-Inh: human C1 esterase inhibitor, CihC: C1-inhibitor and C4bp binding protein, C4bp: C4b-binding protein, CspA: complement regulator-acquiring surface protein-1, CspZ: Complement Regulator-Acquiring Surface Protein 2, DC: dendritic cells, Erp: OspE-F related lipoprotein, FhbA: complement factor H-binding protein, HcpA: human complement regulator and plasminogen binding protein, IL: interleukin, LIC11207: *L. interrogans* serovar Copenhageni (LIC) protein 11207, LigA: Leptospiral immunoglobulin-like protein A, LigB: Leptospiral immunoglobulin-like protein B, LipL32: 32-kDa Lipoprotein of Leptospira, LPS: lipopolysaccharide, LruA: Leptospira interrogans lipoprotein A associated with Recurrent Uveitis, NF-kB: NF-kappa B, NO: nitric oxide, OspA: Outer-surface protein A, OspB: Outer-surface protein B, OspC: Outer-surface protein C, OspE: Outer-surface protein E, PMNs: polymorphonuclear cells, Td: *T. denticola*, TLR: toll-like receptor, TNFa: tumor necrosis factor A, VlsE: variable major protein (VMP)-like sequence E, Vmp: Variable major lipoprotein
